# Clinical and cytokine patterns of uncontrolled asthma with and without comorbid chronic rhinosinusitis: a cross-sectional study

**DOI:** 10.1186/s12931-022-02028-3

**Published:** 2022-05-11

**Authors:** Kai Huang, Fangyuan Li, Xuechen Wang, Bing Yan, Ming Wang, Shuling Li, Wenling Yu, Xiaofang Liu, Chengshuo Wang, Jianmin Jin, Luo Zhang

**Affiliations:** 1 Department of Respiratory and Critical Care Medicine, Beijing Tongren Hospital, Capital Medical University, No. 1, DongJiaoMinXiang, DongCheng District, Beijing, 100730 China; 2 Beijing Key Laboratory of Nasal Diseases, Beijing Institute of Otolaryngology, Beijing, 100005 China; 3 Department of Radiology, Beijing Tongren Hospital, Capital Medical University, Beijing, 100730 China; 4 Department of Otolaryngology Head and Neck Surgery, Beijing Tongren Hospital, Capital Medical University, No. 1, DongJiaoMinXiang, DongCheng District, Beijing, 100730 China; 5 Department of Allergy, Beijing Tongren Hospital, Capital Medical University, Beijing, 100730 China; 6 Research Unit of Diagnosis and Treatment of Chronic Nasal Diseases, Chinese Academy of Medical Sciences, Beijing, China

**Keywords:** Uncontrolled asthma, Clinical patterns, Comorbid chronic rhinosinusitis, Cytokine profile, Cluster analysis

## Abstract

**Background:**

Asthma is significantly related to chronic rhinosinusitis (CRS) both in prevalence and severity. However, the clinical patterns of uncontrolled asthma with and without comorbid CRS are still unclear. This study aimed to explore the clinical characteristics and cytokine patterns of patients with uncontrolled asthma, with and without comorbid CRS.

**Methods:**

22 parameters associated with demographic characteristics, CRS comorbidity, severity of airflow obstruction and airway inflammation, and inflammation type of asthma were collected and assessed in 143 patients with uncontrolled asthma. Different clusters were explored using two-step cluster analysis. Sputum samples were collected for assessment of Th1/Th2/Th17 and epithelium-derived cytokines.

**Results:**

Comorbid CRS was identified as the most important variable for prediction of different clusters, followed by pulmonary function parameters and blood eosinophil level. Three clusters of patients were determined: Cluster 1 (n = 46) characterized by non-atopic patients with non-eosinophilic asthma without CRS, demonstrating moderate airflow limitation; Cluster 2 (n = 54) characterized by asthma patients with mild airflow limitation and CRS, demonstrating higher levels of blood eosinophils and immunoglobulin E (IgE) than cluster 1; Cluster 3 (n = 43) characterized by eosinophilic asthma patients with severe airflow limitation and CRS (46.5% with nasal polyps), demonstrating worst lung function, lowest partial pressure of oxygen (PaO_2_), and highest levels of eosinophils, fraction of exhaled nitric oxide (FeNO) and IgE. Sputum samples from Cluster 3 showed significantly higher levels of Interleukin (IL)-5, IL-13, IL-33, and tumor necrosis factor (TNF)-α than the other two clusters; and remarkably elevated IL-4, IL-17 and interferon (IFN)-γ compared with cluster 2. The levels of IL-10 and IL-25 were not significantly different among the three clusters.

**Conclusions:**

Uncontrolled asthma may be endotyped into three clusters characterized by CRS comorbidity and inflammatory cytokine patterns. Furthermore, a united-airways approach may be especially necessary for management of asthma patients with Type 2 features.

**Supplementary Information:**

The online version contains supplementary material available at 10.1186/s12931-022-02028-3.

## Background

Asthma is a heterogeneous disease, with different underlying processes. Recognizable clusters of demographic, clinical and/or pathophysiological characteristics are often referred to as “asthma phenotypes”. However, no strong association has been found between specific pathological features and particular clinical patterns or treatment responses [1]. Thus, endotype analysis of asthma focused on distinct pathophysiological mechanism is likely to be important, as it will point physicians towards more personalized management [2, 3].

An increasing number of studies have provided consistent evidence that asthma is closely related to chronic rhinosinusitis (CRS), thus strongly supporting the united airway concept [4, 5]. Indeed, about 50% of patients with CRS have comorbid asthma, whereas 40 ~ 75% of patients with asthma have comorbid CRS [3, 6]. Although there is no direct evidence of causality between the two conditions, a direct correlation has nevertheless been found between CRS severity and asthma severity [4, 7]. Although a recent study by Wu and colleagues [8] has demonstrated that the clinical phenotypes of patients with both nasal polyps and comorbid asthma (NPcA) could be divided into 3 clusters, mainly according to onset, duration and severity of symptoms, studies investigating phenotypes of asthma based on CRS comorbidity, severity of airflow obstruction and airway inflammation, and inflammation type of asthma using cluster analysis are scarce.

Investigation of airway inflammatory characteristics, especially the expression of proinflammatory cytokines in patients with uncontrolled asthma is meaningful, as the feature of airway inflammation may be more prominent in the uncontrolled stage, and biologics targeting the related cytokines can be adopted more precisely for treatment of uncontrolled asthma. Since both Th2 and non-Th2 mechanisms are involved in asthma pathogenesis, simultaneous evaluations of traditional Th1/Th2/Th17 and related epithelium-derived cytokines in the airway are necessary. Seys and colleagues [9] evaluated sputum cytokine mRNA expression in an unselected group of adults with stable asthma, and demonstrated that higher levels of IL-5, IL-17A and IL-25 in the airways were associated with uncontrolled asthma and worse lung function. Hasegawa and colleagues [10] have recently shown that serum levels of IL-17A were significantly different between uncontrolled and well-controlled patients, and that IL-17A levels were correlated with levels of IL-4, IL-25, IL-10, and IFN-γ in patients with uncontrolled asthma. Although, the patients with the highest serum levels of all these cytokines manifested refractory asthma, the clinical and airway inflammatory characteristics of these patients were not further investigated [10]. To date, the airway expression patterns of traditional Th1/Th2/Th17 and related epithelium-derived cytokines have not been fully clarified in the specific phenotypes of patients with asthma.

Ideal criteria for defining heterogeneity of asthma are recommended to be based on both phenotype and putative pathophysiology (endotype) [3, 5]. In the present study, we have defined heterogeneity of uncontrolled asthma based on cluster analysis of a combination of clinical characteristics in the united-airways, and sputum “inflammatory cytokine patterns”. Briefly, different clusters were first explored using two-step cluster analysis, according to comorbidity of CRS, severity of airflow obstruction and airway inflammation, and inflammation type of asthma. Subsequently, Th1/Th2/Th17 cytokines and related epithelium-derived cytokines were measured in sputum samples to investigate if patients in a particular cluster shared a common inflammatory mechanism.

## Methods

### Subjects and study protocol

This was a single center cross-sectional study, involving patients with uncontrolled asthma. Patients referred to Beijing Tongren Hospital for complaints of recurrent and intermittent wheeze, shortness of breath, chest tightness, and cough were consecutively enrolled into the study from Sept 2017 to Sept 2019.

Asthma and uncontrolled asthma were defined according to the criteria of Global Initiative for Asthma (GINA) [11]. Briefly, asthma was diagnosed based on history of variable respiratory symptoms (wheeze, shortness of breath, chest tightness and cough), variable expiratory airflow limitation confirmed by spirometry (positive bronchodilator (BD) reversibility test/positive bronchial challenge test/significant increase in lung function after 4 weeks of anti-inflammatory treatment), and exclusion of other conditions that mimic asthma. The variable airflow limitation was determined based on the results of both lung function measurement on enrollment and previous results, as most of the patients were previously diagnosed as asthmatic. In this study, BD reversibility test was positive at least once in 121 patients, bronchial challenge test was positive at least once time in 15 patients, and significant increase in lung function after 4 weeks of anti-inflammatory treatment was found in 12 patients. At enrollment, all but five patients were found to have been diagnosed as asthma patients for at least 3 months; with the five patients being newly diagnosed as asthmatic, based on typical symptoms manifestation and results of lung function tests. Moreover, the diagnosis was further confirmed during follow up. Asthma control was evaluated based on the following 4 items: (1) Daytime asthma symptoms more than twice/week? (2) Any night waking due to asthma? (3) Reliever needed for symptoms more than twice/week? (excluding reliever taken before exercise) (4) Any activity limitation due to asthma? Uncontrolled asthma was defined if at least 3 of these 4 items were prevailing during the week before enrollment, regardless of the medication status [11, 12]. Chronic rhinosinusitis (CRS) was diagnosed based on the European Position Paper on Rhinosinusitis and Nasal Polyps (EP3OS) criteria by clinical symptoms and features of sinus computed tomography (CT) [13]: (1) For the clinical diagnosis of sinusitis, major and minor criteria were adopted. Major symptoms included nasal obstruction/blockage and nasal or post-nasal discharge/purulence. Minor symptoms were facial pain/pressure/fullness, headache, and hyposmia/anosmia. Patients were classified as having CRS if they reported usual presence of at least two of these symptoms for 12 consecutive weeks, and at least one of the two major symptoms was present. (2) CT features included mucosal changes of sinuses and/or ostiomeatal complex. The presence/absence of nasal polyps (NP) was ascertained by nasal endoscopy performed by two experienced otolaryngologists specifically assigned to do this, and any differences in the diagnoses by the otolaryngologists were resolved by discussion and their final consensus. All patients were aged ≥ 18 years, and patients were excluded from the study if they met any of the following criteria: (i) smoking index > 10 pack·year, (ii) received systemic steroid therapy in the proceeding 4 weeks, (iii) received any other immunosuppressive therapy or biologics treatment, (iv) diagnosed with other lung disease such as chronic obstructive pulmonary disease, pneumonia, lung cancer, allergic bronchopulmonary aspergillosis, active pulmonary tuberculosis and interstitial lung disease; (v) diagnosed with immunodeficiency or autoimmune disease, (vi) diagnosed with gastroesophageal reflux disease or parasite infection, and vii) with severe heart failure or significant dysfunction of other organs. Similarly, patients with fungal sinusitis, inverted papilloma, or other nasal diseases that could affect results of the study were also excluded. Additionally, patients with a post-bronchodilator FEV1/FVC less than 0.7 were required to have no history of smoking, no exposure to burning of wood and other biomass fuels, no occupational exposure to dusts and chemical agents, and no definite lung hyperinflation on CT images, in order to exclude COPD comorbidity as much as possible.

**Fig. 1:**
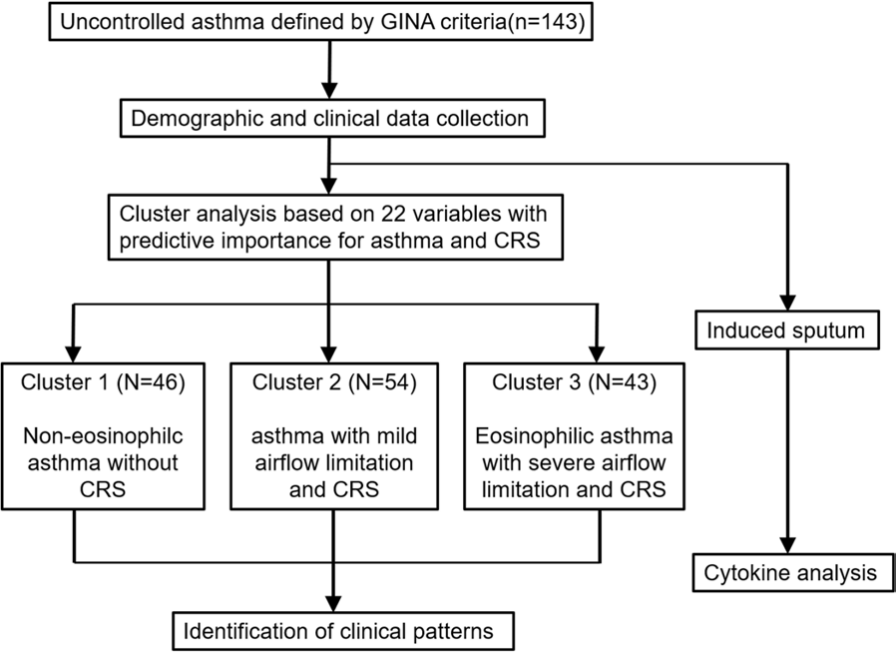
Study flowchart. *GINA* global initiative for asthma, *CRS* chronic rhinosinusitis

On enrollment, demographic and clinical data were collected for each patient using standard questionnaires, medical histories, and other records; including general demographics, age at first symptoms of asthma, nasal disease and other underlying disease, and medication use for asthma. Blood and sputum were collected for further analyses, and the patients were examined for various clinical parameters as detailed below. 22 parameters associated with demographic characteristics (gender, age, body mass index [BMI], asthma onset age), comorbid CRS (comorbidity of CRS/NP, Lund-Mackay score [LMS]), severity of airflow obstruction and airway inflammation (pulmonary function parameters, blood gas analysis, thickness of bronchial wall, comorbidity of bronchiectasis), and inflammation type of asthma (atopy, immunoglobulin E [IgE] level, blood/sputum eosinophil [EOS] level, fractional exhaled nitric oxide [FeNO]), were collected and clustered using two-step cluster analysis. Sputum samples from a number of patients in each cluster were subsequently analyzed for several cytokines to investigate the possible mechanism/s of airway inflammation. The study flow chart is shown as Fig. 1.

The study was approved by the local ethics committee of Beijing Tongren Hospital, Capital Medical University, and written informed consent was obtained from all patients prior to entry into the study.

### Detection of serum total (T)-IgE and allergen specific (s)-IgE

An automatic immunoassay system (ImmunoCap TM100, Pharmacia Company, Sweden) was used to assess serum T-IgE and s-IgE, according to the manufacturer′s directions. The lower limit of detection of serum T-IgE and allergen s-IgE were 2 kU/L and 0.01 kUA/L, respectively. Serum T-IgE > 60 kU/L and allergen specific IgE > 0.35 kUA/L were considered abnormally elevated [14, 15].

### Determination of atopic status

Atopy was assessed by serum levels of specific IgE (s-IgE) generated to a panel of common aeroallergens (dust mites, pollen, cat, dog, cockroach, molds) and food allergens (albumen, milk, peanut, meat, seafood). Atopy was defined when any of the serum s-IgE tested was > 0.35 kUA/L [14].

### Fractional exhaled nitric oxide test

Fractional exhaled nitric oxide (FeNO) was measured using a nitric oxide analyzer (Niox; Aerocrine, Solna, Sweden) at a flow rate of 50 mL/s via the oral cavity. The exhaled gas was continuously routed into the analyzer via a side port. The procedure was repeated three times and a mean concentration of FeNO was calculated [15].

### Pulmonary function test

Spirometry (JAEGER, MasterScreen-body + diffusion + APS, Germany) was performed to determine the lung function measurements and bronchodilator reversibility. Pre- and Post-bronchodilator forced expiratory volume in 1 s (FEV_1_)/forced vital capacity (FVC)%, and FEV_1_%predicted (FEV1%pred) were measured before bronchodilator (BD) treatment and 15 min after inhalation of 400 μg salbutamol, respectively.

### Blood gas analysis

Samples of arterial blood (2 ml) were withdrawn during normal air breathing and assessed for partial pressure of oxygen (PaO_2_) and partial pressure of carbon dioxide (PaCO_2_) using a blood gas analyzer [GEM Premier 3000, United States]. Blood gas analysis was completed within 5 min after collection of the blood sample.

### Peripheral eosinophil count

Blood samples were drawn in vacutainer tubes with EDTA as anticoagulant (Ref. 368274, Becton Dickinson, USA). Leukocyte count and eosinophil (EOS) differentiation in anticoagulated blood samples were performed on an automatic blood analyzer (sysmex XN10B3, Japan).

### Chest CT, determination of bronchiectasis and bronchial wall evaluation

High resolution computed tomography (HRCT) of the chest was performed using a 64-row, multiple-detector CT scanner (Philips Company, Netherlands). An experienced radiologist blinded to the clinical and laboratory data reviewed the CT scans. A diagnosis of bronchiectasis on chest HRCT was made if bronchial wall thickening was present and the ratio of the diameter of bronchus to that of the accompanying pulmonary artery was greater than 1.1 (signet ring sign), or there was lack of tapering of bronchi (tramline sign). Mild bronchiectasis only visible in a single pulmonary segment was not taken into consideration, as this may exist in a significant percentage of healthy population [16]. Bronchial wall thickness was evaluated based on chest HRCT according to the method described by Awadh and colleagues [17]. Both the ratio of airway wall thickness to total diameter (the T/D ratio) and the percentage wall area (WA%) were used to evaluate airway wall thickening.

### Sinus CT and evaluation of CRS

Sinus CT was performed using a 64-row, multiple-detector CT scanner (Philips Company, Netherlands). The radiographic severity of CRS was assessed, by an experienced radiologist blinded to the clinical and laboratory data, using the Lund-Mackay CT staging system (0–24) [18]. The Lund-Mackay score (LMS) of “0” was considered as normal or negative, whereas a score ≥ 1 was considered as abnormal or positive.

### Sputum induction, eosinophil count and cytokine measurement

Sputum induction was performed in all the patients and the samples were prepared for routine clinical examination, according to the method described before [19]. Briefly, the sputum samples were kept on ice and processed within 1 h. Sputum was weighed and mixed with 0.1% dithiothreitol in a ratio of 4:1. The sample was then filtered through 48-μm gauze to increase homogenization and centrifuged at 1,500 × g for 15 min at 4 °C. The supernatants from the sputum samples of the final 23 patients enrolled in this study (5 from cluster 1, 9 from cluster 2, and 9 from cluster 3) were harvested and stored at -80 °C until analysis for cytokines; including IL-4, IL-5, IL-13, IL-25, IL-33, IFN-γ, TNF-α, IL-10 and IL-17; using the Luminex assay (Luminex, Austin, Tex). The cell pellets were used to prepare cytospin slides according to Brightling and colleagues [20], and then stained with Wright Giemsa stain for assessment of eosinophil counts. The stained slides were examined by an experienced observer, who was blinded to the patient’s clinical data, to obtain a differential cell count by counting 400 non-squamous cells.

## Medication on enrollment

The medications used by the patients within 72 h before enrollment were recorded. No usage of inhaled corticosteroids (ICS), long-acting beta_2_-agonist (LABA), leukotriene receptor antagonist (LTRA), long-acting muscarinic anticholinergics (LAMA), theophylline and anti-histamines; but not drugs such as short-acting beta_2_-agonist (SABA); within 72 h before enrollment was recorded as “No medication”.

### Assessment of asthma severity

Asthma severity was assessed during follow-up using the stepwise approach for adjusting asthma treatment in adults, the GINA criteria [11]. Thus, mild asthma was defined as asthma that was well controlled with Step 1 or Step 2 treatment; moderate asthma was defined as asthma that was well controlled with Step 3 treatment; and severe asthma was defined as asthma that required Step 4 or Step 5 treatment.

### Statistical analysis

Statistical analyses were performed using the SPSS 23.0 statistical software package (Statistics Package for the Social Sciences, SPSS 23.0, Inc., Chicago, IL, USA). A two-step cluster analysis was used to identify homogeneous groups of cases which were not previously known, based on a combination of different types of variables; Schwarz’s Bayesian Criterion (BIC) was chosen in the clustering criterion [21]. Data were expressed as mean ± standard deviation (for normally distributed parameters) or as median and 25th-75th percentiles (for non-normally distributed parameters). Comparisons of continuous data between the clusters were performed by ANOVA (for normally distributed parameters) and by Kruskal Wallis Test (for non-normally distributed parameters). Categorical variables between different clusters were analyzed by χ^2^ test. *P* values less than 0.05 were considered as statistically significant. For multiple comparisons among three clusters (for χ^2^ test and Kruskal Wallis Test), *P* values less than 0.017 were considered as statistically significant.

## Results

### Demographic and clinical characteristics of the study population

The study cohort consisted of 143 patients with uncontrolled asthma, of whom 50 were male (34.9%) and 93 were female (65.1%), with a mean age of 55 (20 ~ 90) years and BMI of 24.7 kg/m^2^. Overall, 87.5% (125/143) of patients had an asthma onset age ≥ 18 years; with the mean age of asthma onset being 39.8 years. A total of 67.1% of patients had comorbid CRS and 16.8% patients comorbid NP, and a mean Lund-Mackay score of 7. Blood gas analysis demonstrated mean PaO_2_ and median PaCO_2_ values of 76.9 mmHg and 40 mmHg, respectively; the median FEV_1_/FVC values before and after BD were 72.4% and 74.2%, and the mean of FEV_1_% predicted before and after BD were 72.5%, and 78.6% respectively. Bronchiectasis was present in 18.9% of the patients; the average values of T/D ratio and WA% were 0.248 and 0.736, respectively. A total of 46.1% patients demonstrated atopy (total serum IgE = 137kU/L), and blood EOS percentage, blood EOS count and FeNO values of 4.8%, 0.32 × 10^9^/L and 39.2 ppb respectively. Sputum eosinophils were found in only 24.4% (35/143) of the patients. Majority of the patients were using inhaled corticosteroids (ICS; 65%) and long-acting beta2-agonist (LABA; 54.5%), whereas 20.9% of patients were not on the specified medication at enrollment (Table 1).

**Table 1:** Comparison of characteristics within the 3 clusters

Parameters	Total [n, (%)]	Cluster 1 [46, (32.2)]	Cluster 2 [54, (37.8)]	Cluster 3 [43, (30.0)]	*p* value
Demographic characteristics					
Age (years)^‡^	55.0 (42.0, 64.0)	59.0 (48.5, 66.2)	54.5 (43.7, 65.0)	47.0 (39.0, 61.0)	0.033*
Male, n (%)	50 (34.9)	13 (28.3)	13 (24.1)	24 (55.8)	0.003*
Asthma onset age (years)^†^	39.8 (17.6)	41.6 (21.0)	40.3 (17.5)	37.2 (13.4)	0.338
BMI (kg/m^2^)^‡^	24.7 (22.3, 27.1)	24.9 (23.2, 27.5)	24.5 (22.5, 26.1)	24.4 (21.0, 27.3)	0.484
Characteristics associated with comorbid CRS					
Comorbid CRS, n (%)	96 (67.1)	1 (2.2)	54 (100.0)	41 (95.3)	< 0.001*
Comorbid NP, n (%)	24 (16.8)	0 (0.0)	4 (7.4)	20 (46.5)	< 0.001*
Lund-Mackay score^‡^	7 (0, 14)	0 (0, 0)	8 (6, 12)	16 (10, 20)	< 0.001*
Severity of airflow obstruction and airway inflammation					
Blood gas analysis					
PaO_2_ (mmHg)^†^	76.9 (11.0)	78.8 (10.2)	80.0 (12.0)	71.0 (8.0)	< 0.001*
PaCO_2_ (mmHg)^‡^	40.0 (37.0, 42.0)	40.0 (37.0, 42.0)	38.0 (35.0, 40.2)	41.0 (39.0, 44.0)	< 0.001*
Spirometry					
FEV1/FVC before bronchodilator (%)^‡^	72.4 (59.1, 83.7)	78.1 (64.5, 85.1)	82.6 (75.8, 86.4)	56.7 (50.5, 62.0)	< 0.001*
FEV1/FVC after bronchodilator (%)^‡^	74.2 (63.0, 83.8)	74.5 (65.2, 85.9)	83.1 (78.6, 87.2)	60.9 (53.6, 65.1)	< 0.001*
FEV_1_%pred before bronchodilator (%)^‡^	72.5 (23.7)	73.4 (20.6)	88.7 (16.5)	51.2 (17.2)	< 0.001*
FEV_1_%pred after bronchodilator (%)^†^	78.6 (21.9)	78.6 (18.8)	92.6 (14.2)	60.9 (17.7)	< 0.001*
Chest CT					
Bronchiectasis, n (%)	27 (18.9)	6 (13.0)	9 (16.6)	12 (27.9)	0.175
T/D ratio^†^	0.248 (0.187)	0.248 (0.019)	0.242 (0.017)	0.254 (0.017)	0.005*
WA%^†^	0.736 (0.389)	0.737 (0.396)	0.726 (0.037)	0.750 (0.036)	0.009*
Characteristics associated with inflammation type					
Atopy, n (%)	66 (46.1)	14 (30.4)	22 (40.7)	30 (69.8)	0.001*
Blood eosinophil percentage (%) ^‡^	4.8 (2.6, 9.5)	2.6 (1.8, 4.9)	4.4 (2.4, 8.9)	9.9 (5.0, 13.0)	< 0.001*
Blood eosinophil count (× 10^9^/L)^‡^	0.32 (0.15, 0.59)	0.16 (0.11, 0.29)	0.32 (0.14, 0.58)	0.61 (0.38, 0.90)	< 0.001*
Serum T-IgE (kU/L)^‡^	137.0 (47.6, 312.0)	56.2 (22.5, 173.0)	142.0 (46.8, 329.0)	213.0 (133.0, 533.0)	< 0.001*
Log(T-IgE)^‡^	2.07 (0.62)	1.80 (0.60)	2.06 (0.60)	2.37 (0.54)	< 0.001*
Sputum eosinophil percentage (%)^‡^	0 (0, 0)	0 (0, 0)	0 (0, 0)	0 (0, 2.5)	0.006*
FeNO (ppb)^‡^	39.2 (20.0, 54.3)	33.0 (17.0, 39.8)	39.4 (13.0, 52.2)	54.5 (32.0, 84.0)	< 0.001*
Medication on enrollment					
No medication, n (%)	30 (20.9)	14 (30.4)	11 (20.4)	5 (11.6)	0.092
ICS, n (%)	93 (65.0)	25 (54.3)	32 (59.3)	36 (83.7)	0.008*
LABA, n (%)	78 (54.5)	19 (41.3)	26 (48.1)	33 (76.7)	0.002*
LTRA, n (%)	42 (29.3)	13 (28.3)	18 (33.3)	11 (25.6)	0.693
Theophylline, n (%)	38 (26.5)	13 (28.3)	13 (24.1)	12 (27.9)	0.87
Antihistamines, n (%)	20 (14.0)	3 (6.5)	10 (18.5)	7 (16.3)	0.198
LAMA, n (%)	0	0	0	0	–
Asthma severity^§^					< 0.001*
Mild, n (%)	66 (49.6)	24 (57.1)	32 (66.7)	10 (23.3)	< 0.001*
Moderate, n (%)	46 (34.6)	14 (33.3)	14 (29.2)	18 (41.9)	0.436
Severe, n (%)	21 (15.8)	4 (9.5)	2 (4.2)	15 (34.9)	< 0.001*

BMI, body mass index; CRS, chronic rhinosinusitis; NP, nasal polyps; PaO_2_, partial pressure of oxygen; PaCO_2_, partial pressure of carbon dioxide; T-IgE, total IgE; FeNO, fractional exhaled nitric oxide; ppb, parts per billion; CT, computed tomography; T/D ratio, ratio of airway wall thickness to total diameter; WA%, percentage of airway wall area to total area; ICS, inhaled corticosteroids; LABA, long-acting beta2-agonist; LTRA, Leukotriene receptor antagonist; LAMA, long-acting muscarinic anticholinergics. *statistical significance. ^†^values are presented as means (SD). ^‡^values are presented as medians (25–75% interquartile range).^§^Totally 133 patients were assessed for asthma severity, including 42 patients in cluster 1, 48 in cluster 2, and 43 in cluster 3

### Cluster analysis of the subjects

**Fig. 2:**
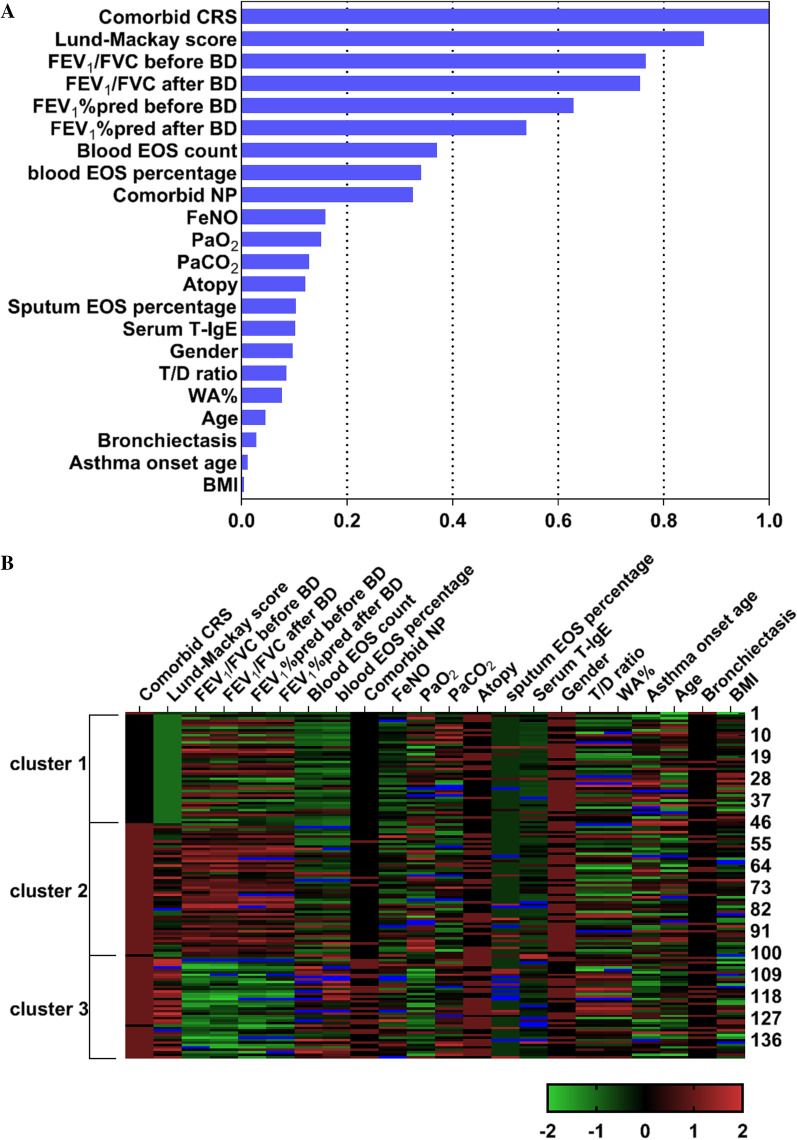
Two-step clustering of 22 demographic and clinical variables. **A** Predictive importance of the variables. **B** Heat map of clustering characteristics in uncontrolled asthma. Each row represents one patient, and the range is shown in each column; with green being the lowest and red the highest. Left: the three clusters are indicated by vertical bars; Right: the number of patients. *CRS* chronic rhinosinusitis, *FEV*_*1*_ forced expiratory volume in 1 s, *FVC* forced vital capacity, *BD* bronchodilator, *EOS* eosinophil, *NP* nasal polyps, *FeNO* fractional exhaled nitric oxide, *PaO*_*2*_ partial pressure of oxygen, *PaCO*_*2*_ partial pressure of carbon dioxide, *T-IgE* total IgE, *T/D ratio* ratio of airway wall thickness to total diameter, *WA%* percentage of airway wall area to total area, *BMI* body mass index

The predictive importance of 22 demographic and clinical variables included in the cluster analysis is shown in Fig. 2A; with CRS comorbidity found to be the most important parameter for clustering, followed by pulmonary function parameters and blood EOS level. Three clusters with distinct characteristics were identified based on these variables (Table 1, Fig. 2B). Cluster 1 (n = 46) was characterized by non-eosinophilic asthma without CRS, with moderate airflow limitation, and blood EOS count < 300/µl in 80% (37/46) of the patients; Cluster 2 (n = 54) was characterized with patients having mild airflow limitation and CRS, with blood EOS counts ≥ 300/µl in 53.7% (29/54) of the patients; Cluster 3 (n = 43) was characterized with patients having eosinophilic asthma with severe airflow limitation and CRS**,** with blood EOS counts ≥ 300/µl in 86% (37/43) of the patients.

### Comparison of characteristics within the 3 clusters

#### Comparison of demographic characteristics within the 3 clusters

There were significant differences in age (*P* = 0.033) and sex ratio (*P* = 0.003) within the 3 clusters. The patients in cluster 3 were significantly younger than in cluster 1 (*P* = 0.011); however, the patients in these 2 clusters were not significantly different in age compared to those in cluster 2 (*P* = 0.336 and 0.079, respectively). The proportion of males in cluster 3 was higher than in cluster 1 (*P* = 0.008) and cluster 2 (*P* = 0.001), with no difference between clusters 1 and 2 (*P* = 0.634). The three clusters were not significantly different with regards to asthma onset age (*P* = 0.338) or BMI (*P* = 0.484) (Table 1).

#### Comparison of characteristics associated with comorbid CRS within the 3 clusters

Striking differences in comorbidity of both CRS (*P* < 0.001) and NP (*P* < 0.001) were seen within the 3 clusters. Presence of comorbid CRS in cluster 1(2.2%) was much lower than in cluster 2 (100%) and cluster 3 (95.3%) (both *P* < 0.001), with no statistical difference between clusters 2 and 3 (*P* = 0.378). The presence of comorbid NP in cluster 3 (46.5%) was significantly higher than in cluster 1 (0%) and cluster 2 (7.4%) (both *P* < 0.001), with no statistical difference between clusters 1 and 2 (*P* = 0.170). A notable difference was also found in the Lund-Mackay score (LMS) within the 3 clusters (*P* < 0.001). The LMS was highest in cluster 3 and lowest in cluster 1, with significant differences between any two clusters. (*P* < 0.001 for all) (Table 1, Additional file 1: Fig. S1A, B).

#### Comparison of severity of airflow obstruction and airway inflammation within the 3 clusters

There were statistical differences in PaO_2_ and PaCO_2_ (both *P* < 0.001) within the 3 clusters (Table 1). The PaO_2_ level in cluster 3, especially in the patients with comorbid chronic rhinosinusitis with nasal polyps (CRSwNP), was much lower than that in cluster 1 (*P* < 0.001) and cluster 2 (*P* < 0.001), with no significant difference between clusters 1 and 2 (Additional file 1: Fig. S2A). The PaCO_2_ level in cluster 2 was significantly lower than that in cluster 1 (*P* = 0.008) and cluster 3 (*P* < 0.001), with no significant difference between clusters 1 and 3 (Additional file 1: Fig. S2B). In particular, striking differences were found in FEV_1_/FVC and FEV_1_%pred within the 3 clusters, either before or after bronchodilator (*P* < 0.001 for all) (Table 1). FEV_1_/FVC and FEV_1_%pred, values either before or after bronchodilator, were the best in cluster 2 and the poorest in cluster 3, with significant differences between any two clusters (FEV_1_/FVC% before bronchodilator: *P* = 0.015, *P* < 0.001, and *P* < 0.001, respectively; FEV_1_/FVC% after bronchodilator: *P* = 0.004, *P* < 0.001, and *P* < 0.001, respectively; FEV_1_%pred before bronchodilator: *P* < 0.001 for all; FEV_1_%pred after bronchodilator: *P* < 0.001 for all) (Additional file 1: Fig. S2C–F). Based on analysis of chest HRCT, significant differences were found in the values of both T/D (*P* = 0.005) and WA% (*P* = 0.009), but no difference was seen in the prevalence of bronchiectasis within the 3 clusters (Table 1). Both T/D and WA% were remarkably higher in cluster 3 than in cluster 2 (*P* = 0.001 and *P* = 0.002, respectively), but not significantly different between cluster 1 and 2 (*P* = 0.123 and *P* = 0.158, respectively), or between cluster 1 and 3 (*P* = 0.102 and *P* = 0.107, respectively) (Additional file 1: Fig. S2G–I).

#### Comparison of characteristics associated with inflammation type within the 3 clusters

Significant differences were found in atopy status (*P* = 0.001), serum T-IgE (*P* < 0.001), log (T-IgE) (*P* < 0.001), blood EOS count (*P* < 0.001), blood EOS percentage (*P* < 0.001), sputum EOS percentage (*P* = 0.006), and FeNO (*P* < 0.001) within the 3 clusters (Table 1). The proportion of atopy in cluster 3 was higher than that in cluster 1 (*P* < 0.001) and cluster 2 (*P* = 0.004), with no difference between clusters 1 and 2 (*P* = 0.285). Serum T-IgE was elevated in cluster 3 compared with cluster 1 (*P* < 0.001), but not significantly different between clusters 1 and 2 (*P* = 0.028), or between cluster 2 and 3 (*P* = 0.033). However, the value of log (T-IgE) was the highest in cluster 3 and the lowest in cluster 1, with significant differences between any two clusters (*P* < 0.001, *P* = 0.011, *P* = 0.027, respectively) (Additional file 1: Fig. S3A). In particular, the blood EOS count and blood EOS percentage were most elevated in cluster 3, and significantly higher compared to the respective values in clusters 1 (both *P* < 0.001) and 2 (both *P* < 0.001); and the levels of eosinophils in cluster 2 were significantly higher than those in cluster 1 (*P* = 0.003 and *P* = 0.008, respectively) (Additional file 1: Fig. S3B, C). Similarly, the value of FeNO in cluster 3 was found to be the highest within the 3 clusters, and significantly different compared with clusters 1 (*P* < 0.001) and 2 (*P* = 0.002) (Additional file 1: Fig. S3D). The sputum EOS percentage was also higher in cluster 3 compared with that in cluster 1 (*P* = 0.002), but not statistically different between clusters 1 and 2, or clusters 2 and 3 (Additional file 1: Fig. S3E).

#### Comparison of medications between the 3 clusters at enrollment

Significant difference was noted in the proportion of patients using inhaled corticosteroids (ICS, *P* = 0.008) or long-acting β_2_-receptor agonists (LABA, *P* = 0.002) (Table 1). The proportion of patients taking ICS or LABA in cluster 3 was much higher compared with that in clusters 1 (*P* = 0.003, *P* = 0.009, respectively) and 2 (*P* = 0.001, *P* = 0.004, respectively); however, no statistical differences were found within the three clusters in the proportions of patients taking no medication, taking leukotriene receptor antagonists (LTRA), theophylline, or antihistamines (Table 1).

#### Comparison of asthma severity within the 3 clusters

A total of 133 patients were assessed during follow-up for asthma severity; including 42 patients in cluster 1, 48 in cluster 2, and 43 in cluster 3. There was statistical difference in asthma severity within the 3 clusters (*P* < 0.001) (Table 1); with significant differences found in the proportions of mild and severe asthma among the 3 clusters (both *P* < 0.001). The difference in the proportion of moderate asthma among the 3 clusters were not significant (*P* = 0.436). The highest proportion of severe asthmatics and the lowest proportion of mild asthmatics were found in cluster 3, when compared with those in cluster 1 (*P* = 0.001, *P* = 0.005, respectively) and 2 (both *P* < 0.001). No significant difference in asthma severity was found between cluster 1and 2 (*P* = 0.493).

#### Comparison of sputum cytokines within the 3 clusters

**Fig. 3:**
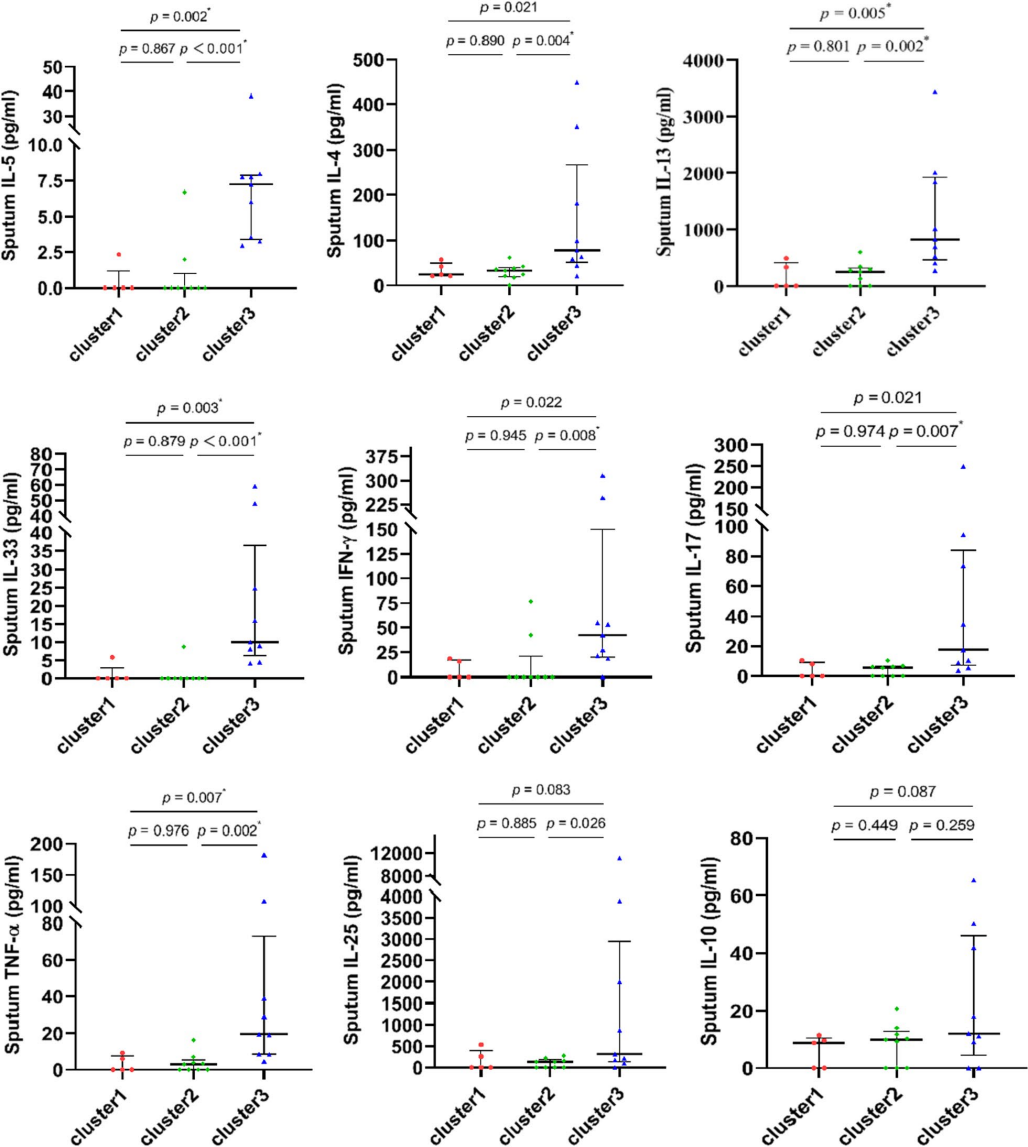
Pairwise comparison of cytokines in sputum samples of patients in the three clusters. *statistical significance

Analysis of induced sputum samples collected from 23 patients; including 5 samples from cluster 1, 9 samples from cluster 2 and 9 samples from cluster 3; demonstrated that the levels of IL-5 (*P* = 0.007), IL-4 (*P* = 0.001), IL-13 (*P* = 0.002), IL-33 (*P* < 0.001), IFN-γ (*P* = 0.013), TNF-α (*P* = 0.002), and IL-17 (*P* = 0.012) were significantly different within the 3 clusters. However, no differences were found in the levels of IL-10 or IL-25 within the 3 clusters (*P* = 0.28 and *P* = 0.058, respectively) (Table 2). The increase in sputum cytokines was most prominent in cluster 3, with statistical differences compared with cluster 1 (for IL-5, IL-13, IL-33, and TNF-α; *P* = 0.002, *P* = 0.005, *P* = 0.003, *P* = 0.007, respectively) and cluster 2 (for IL-4, IL-5, IL-13, IL-33, IFN-γ, TNF-α and IL-17; *P* = 0.004, *P* < 0.001, *P* = 0.002, *P* < 0.001, *P* = 0.008, *P* = 0.002, and *P* = 0.007, respectively). Although the levels of IL-4, IFN-γ and IL-17 were different between cluster 3 and cluster 1, these were not statistically different (*P* = 0.021, *P* = 0.022, and *P* = 0.021, respectively) (Fig. 3).

**Table 2:** Comparison of sputum cytokines within the three clusters

Parameters	Cluster 1 (n = 5)	Cluster 2 (n = 9)	Cluster 3 (n = 9)	*p* value
Male, n (%)	1 (20.0)	1 (11.1)	4 (44.4)	0.257
Age (years)^‡^	55.0 (52.5, 73.5)	63.0 (49.5, 74.0)	62.0 (45.0, 65.5)	0.916
IL-5 (μg/ml)^‡^	0 (0,1.16)	0 (0, 0.98)	7.23 (3.39, 7.85)	< 0.001*
IL-4 (μg/ml)^‡^	24.5 (21.2, 49.6)	32.9 (19.3, 39.9)	78.3 (51.5, 266.9)	0.007*
IL-13 (μg/ml)^‡^	0 (0, 410)	249 (0, 315)	825 (463, 1924)	0.002*
IL-25 (μg/ml)^‡^	0 (0, 399)	133 (0, 186)	317 (138, 2946)	0.058
IL-33 (μg/ml)^‡^	0 (0, 2.92)	0 (0, 0)	10.00 (6.31, 36.49)	< 0.001*
IFN-γ (μg/ml)^‡^	0 (0, 17.1)	0 (0, 21.2)	42.4 (20.0, 150.0)	0.013*
IL-10 (μg/ml)^‡^	8.81 (0, 10.60)	9.88 (0, 12.90)	12.00 (4.59, 46.10)	0.210
IL-17 (μg/ml)^‡^	0 (0, 9.26)	5.66 (0.00, 6.57)	17.60 (7.31, 83.90)	0.012*
TNF-α (μg/ml)^‡^	0 (0, 7.64)	3.01 (0.00, 5.44)	19.60 (8.56, 73.00)	0.002*

*IL* interleukin, *IFN* interferon, *TNF* tumor necrosis factor. *statistical significance. ^‡^values are presented as medians (25–75% interquartile range)

## Discussion

In this cross-sectional study, clinical patterns of uncontrolled asthma were explored, based on cluster analysis of variables with predictive importance for asthma and CRS, and further supported by particular patterns of Th1/Th2/Th17 cytokines in sputum and related epithelium-derived cytokines. Three clinical patterns with distinct characteristics were identified, including non-eosinophilic asthma without CRS [Cluster 1, with least Type 2 (T2) features], asthma with mild airflow limitation and CRS (Cluster 2, with moderate T2 features), and eosinophilic asthma with severe airflow limitation and CRS (Cluster 3, with severe T2 features). Further investigation of sputum cytokines showed that compared with the other 2 clusters, patients in cluster 3, the most severe T2 subgroup, had significantly increased levels of IL-33 and traditional Th1/Th2/Th17 cytokines (especially IL-5, IL-13, TNF-α) in airways, accompanied by relatively deficient production of IL-10. To the best of our knowledge, this is the first study to comprehensively explore clinical patterns of uncontrolled asthma in terms of comorbid CRS, severity of airflow limitation and airway inflammation, inflammatory type of asthma, and sputum profile of Th1/Th2/Th17 and related epithelium-derived cytokines. Furthermore, this is the first study to show that uncontrolled eosinophilic asthma with severe airflow limitation and comorbid CRS (often with NPs) is characterized by enhanced collaborative expression of IL-33 and Th1/Th2/Th17 cytokines in the airways.

The concept “one airway, one disease” maintains that inflammatory processes of the upper and lower airway often co-exist and share common etiopathogenic mechanisms [4]. Asthma combined with CRS is more serious than asthma or CRS alone [22]. Furthermore, based on the phenotype of CRS, i.e. chronic rhinosinusitis without nasal polyps (CRSsNP) and chronic rhinosinusitis with nasal polyps (CRSwNP), it has been shown that asthmatics with comorbid CRSwNP demonstrate more significant airway inflammation and poorer asthma control than asthmatics with CRSsNP [6]. In the current study, asthma and CRS were found to be closely related in terms of prevalence and severity in patients of both clusters 2 and 3, supporting the united airway concept associated with T2 features [3, 5, 12]. Furthermore, these findings suggest that an approach comprising management of a united airway may be especially necessary for asthmatics with T2 features.

The features of mild asthma in cluster 2 and the severe asthma in cluster 3 indicated according to clinical characteristics of patients in the present study were further supported by significant differences in sputum levels of traditional Th1/Th2/Th17 cytokines (IL-5, IL-4, IL-13, IFN-γ, TNF-α, IL-17) and the related epithelium-derived cytokine (IL-33); however, the sputum EOS counts were comparable between these two clusters. This is in accordance with the findings of a recent study from Japan, which demonstrated that the percentages of mixed inflammatory phenotype as well as systemic eosinophilic inflammation were increased in asthma patients with severe CRS; whereas, patients with asthma and mild-moderate CRS mainly demonstrated eosinophilic phenotypes [23]. This is also supported by the findings of Hastie and the colleagues [24], who found that sputum cytokines and chemokines mainly associated with Th1 and Th17 inflammation differed between mild and severe asthma subjects [24]. Although the sputum EOS counts were comparable between clusters 2 and 3, and both were higher than cluster 1, the sputum levels of Th2 cytokines including IL-5, IL-4 and IL-13 in cluster 2 were significantly lower than those in cluster 3, and similar with those in cluster 1. It should be noted that in our study, the sputum samples and analyses characterize patients who had received anti-asthmatic drugs such as ICS therapy, and the airway inflammation may be suppressed to some degree. These findings further support the view that the airway inflammation of patients in cluster 2, with almost normal lung function and less bronchial wall thickness, is sensitive to the therapy; whereas the airway inflammation of patients in cluster 3, with most severe asthma, poorest lung function and most bronchial wall thickness, may be refractory to the therapy. In our study, sputum eosinophil levels of the patients were much lower than those previously reported in white patients [25], and also no definite Th1 or Th2 pattern was found according to sputum cytokines in all the 3 clusters. It may be due to the usage of anti-asthmatic agents, and the ethnic and regional differences of the patients [26]. Although the majority of white patients with CRSwNP in western countries show a type 2/Th2 pattern of inflammation [3, 12, 27], patients in cluster 3 consisting of 46.5% of patients with CRSwNP in the present study demonstrated a Th2/Th1/Th17 pattern. This finding is consistent with the finding from our previous study, which showed Th2 biased inflammation in NP tissues of patients from western countries, and Th2/Th1/Th17mixed patterns in NP tissues of patients from Beijing [26]. However, further studies are needed to clarify the discrepancy in sputum inflammatory cell type and cytokine patterns of patients from different races and regions, and whether or not a common inflammation pattern exists in the upper and lower airways of patients with asthma and comorbid CRSwNP.

Our finding that sputum IL-33, but not IL-25, was significantly higher in cluster 3 compared with clusters 1 and 2, suggests that enhanced T2-biased inflammation in cluster 3 may be regulated by IL-33 rather than IL-25. Although the immunologic functions of IL-25 and IL-33 appear to overlap almost entirely, one large-scale genome-wide association study (GWAS) of asthma has indicated a more pronounced role for IL-33 in asthmatic patients [28]. Furthermore, Barlow and colleagues [29] have demonstrated that the IL-33 pathway is more effective than the IL-25 pathway in regulating airway hyperreactivity (AHR) in mouse models of allergic asthma, while Stolarski and colleagues [30] have shown that IL-33 directly stimulated eosinophil differentiation from CD117 + progenitors in IL-5-dependent manner [30]. One study has indicated that multiple feedback circuits involving IL-33 and type 2 innate lymphoid cells (ILC2s) are necessary for persistence of asthma [31]. These findings may provide clues for adopting anti-IL-33 treatment for patients with severe uncontrolled asthma and comorbid CRS.

Our study suggests that Th1 cytokines (IFN-γ, TNF-α) and Th17 cytokines (IL-17) may cooperate with Th2 cytokines (IL-5, IL-4, IL-13) to aggravate the airway inflammation in patients within cluster 3. Indeed, Th1 cytokines including TNF-α and IFN-γ, have been observed to disrupt the tight junction of human airway epithelium and promote inflammatory cytokine release [3]. TNF-α has been shown to autoregulate its expression and induce adhesion molecule expression in asthma, and contribute to the development of asthma by enhancing IL-23/Th17 and Th2 immune responses [32]. IFN-γ is indicated to cause severe airway inflammation of asthma during symptomatic periods. Kanda and colleagues [33] demonstrated that eosinophil-derived IFN-γ could induce airway hyperresponsiveness and lung inflammation in the absence of lymphocytes [33]. As the patients in cluster 3 manifested as an eosinophilic phenotype, it is tempting to speculate that the airway eosinophils of patients in cluster 3 might secret IFN-γ after enhanced lung recruitment mediated by excessive IL-5, and relatively deficient production of IL-10 may further contribute to the increased airway inflammation. However, this needs to be confirmed by further investigations in the future. Th17 cytokines play an important role in the pathophysiological process of severe asthma [5, 34]. Since IL-17 can amplify selected nuclear factor κB (NF-κB)-dependent signaling pathways induced by TNF-α [35], significant increase in these 2 cytokines may be an underlying mechanism of severe airway inflammation of patients in cluster 3, even with better adherence to ICS treatment [34]. Whilst CD26 has been shown to play a role in down-modulating airway inflammation mediated by Th2 and especially Th17/Th1 cells [28], whether abnormal CD26 expression is involved in the excessive inflammation in patients of cluster 3 needs to be clarified. Nevertheless, Th2 and Th17 inflammatory pathways have been demonstrated to be reciprocally regulated in patients with asthma [35]. Since simultaneous elevations of Th2- (IL-5, IL-4) and Th17-cytokines (IL-17) have been found in sputum and/or serum samples of uncontrolled asthmatics [9, 10], and IL-4 and IL-13 released by IL-33-activated innate lymphoid cells (ILCs) 2 s promote M2 polarization of alveolar macrophages [36, 37], this may implicate involvement of ILCs and alveolar macrophages in the airway inflammation of patients in cluster 3. Indeed, excessive airway IFN-γ may result in M1 polarization of alveolar macrophages [38, 39]; while macrophages, as the main component of inflammatory cells in sputum of asthmatics [40], can secret IL-4, IL-13, TNF-α, IL-17 and IL-10 under different conditions [38–40]. However, the role of these cells in patients in cluster 3 awaits further investigation.

The findings from this study, however, are somewhat limited. The sample size was relatively small, and as a cross-sectional and single-center study, there might be some data deviation and bias of case enrollment. The unicentric nature of the study may also jeopardize the external validity of these results. Although the severity of asthma could be discriminated by the clinical characteristics to a large degree, the status/severity of uncontrolled asthma was not numerically evaluated by asthma control test (ACT) or asthma control questionnaire (ACQ), which would have addressed the potential for significant variations in sputum levels of cytokines. Due to the retrospective nature of the study, exacerbations could not be absolutely excluded, especially for the patients with intermittent symptoms, which were more severe during night, and for patients who suffered from chronic asthma and showed poor perception of symptoms. Also, the percentages of the other inflammatory cell types in sputum sample were not analyzed, and sputum cytokines were detected in small sample of patients. Despite distinct clinical characteristics, no significant differences were found in the traditional Th1/Th2/Th17 and related epithelium-derived cytokines between clusters 1 and 2. Thus, further investigation of the possible inflammatory mechanisms operating in cluster 1 is warranted. In the current study local drug therapy for nasal disease was also not recorded. However, compared with those with mild CRS, patients with severe CRS have been shown to have a better compliance with medication and ask for medical help more often [41].

## Conclusions

Three clinical patterns of uncontrolled asthma were identified; based on united airway concept, cluster analysis of clinical characteristics, and expression patterns of Th1/Th2/Th17 and related epithelium-derived cytokines; which should be helpful for accurate diagnosis and treatment options for patients with uncontrolled asthma. A united-airways approach may be necessary for management of asthmatics with T2 features, especially those with severe comorbid asthma and CRS. Considering the complicated inflammatory network in patients in cluster 3, developing biologics targeting the factor/s (for example, IL-33, IL-5, IL-13, and TNF-α), which may play a central role in the pathophysiology of disease in these patients, should lead to improvement of both the treatment and prognosis of patients with comorbid asthma and CRS. However, further prospective multicentre studies with large sample size will be useful in confirming the findings of this study.

## Supplementary Information

**Additional file 1.** **Figure S1:** Comparison of characteristics associated with comorbid rhinosinusitis within the 3 clusters.

## Data Availability

The complete dataset is included in this manuscript.

## Abbreviations

ACQ: Asthma control questionnaire
ACT: Asthma control test
AHR: Airway hyperreactivity
BD: Bronchodilator
BMI: Body mass index
CRS: Chronic rhinosinusitis
CRSsNP: Chronic rhinosinusitis without nasal polyps
CRSwNP: Chronic rhinosinusitis with nasal polyps
CT: Computed tomography
EOS: Eosinophil
EP3OS: European position paper on rhinosinusitis and nasal polyps
FeNO: Fractional exhaled nitric oxide
FEV1: Forced expiratory volume in 1 s
FVC: Forced vital capacity
GINA: Global initiative for asthma
GWAS: Genome-wide association study
HRCT: High resolution computed tomography
ICS: Inhaled corticosteroids
IFN: Interferon
IgE: Immunoglobulin E
IL: Interleukin
ILCs: Innate lymphoid cells
ILC2s: Type 2 innate lymphoid cells
LABA: Long-acting beta_2_-agonist
LAMA: Long-acting muscarinic anticholinergics
LMS: Lund-Mackay score
LTRA: Leukotriene receptor antagonist
NPcA: Nasal polyps and comorbid asthma
NP: Nasal polyps
PaCO_2_: Partial pressure of carbon dioxide
PaO_2_: Partial pressure of oxygen
ppb: Parts per billion
s-IgE: Specific IgE
T2: Type 2
T/D ratio: Ratio of airway wall thickness to total diameter
Th2: T helper 2
T-IgE: Total IgE
TNF: Tumor necrosis factor
WA%: Percentage of airway wall area to total area
