# Supplementary material for: Clinical and cytokine patterns of uncontrolled asthma with and without comorbid chronic rhinosinusitis: a cross-sectional study
Source: Respir Res. 2022 May 11;23:119. doi: 10.1186/s12931-022-02028-3 (PMC9092818; doi:10.1186/s12931-022-02028-3)

**Supplementary figures**

**Figure S1:** (A) Pairwise comparison of Lund-Mackay score within the three clusters. *statistical significance. (B) Typical sinus CT scans demonstrating the severity of sinusitis within the three clusters; cluster 1: no sinusitis (LMS = 0); cluster 2: moderate sinusitis (LMS = 8); cluster 3: more severe sinusitis (LMS = 23). LMS: Lund-Mackay score.

**A**
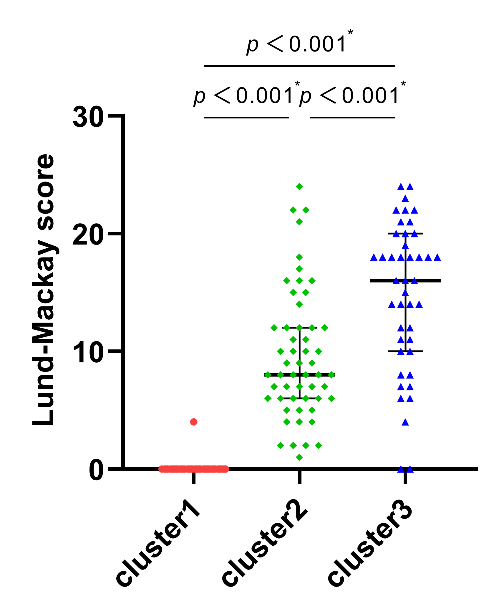


**
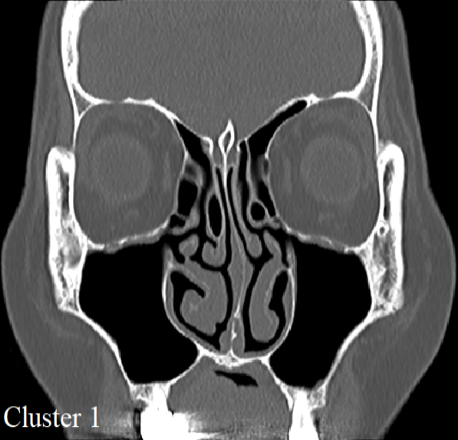

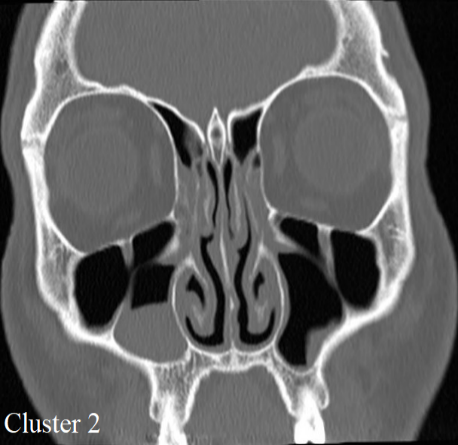

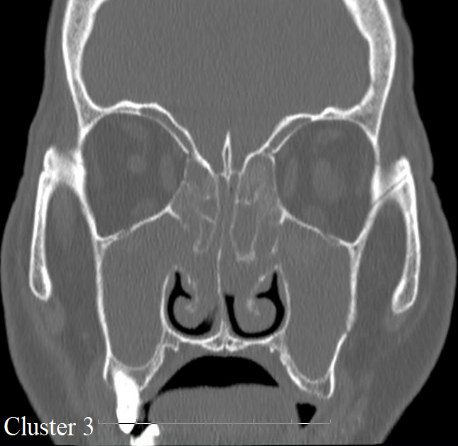
B**

**Figure S2:** (A- H) Pairwise comparison of parameters associated with airflow limitation and airway inflammation within the three clusters. (I) Typical chest high resolution computed tomography (HRCT) scans demonstrating bronchial wall thickening within the three clusters; bronchial wall thickening was greater in cluster 3 (T/D: 0.276; WA%: 0.792) than in cluster 1 (T/D: 0.265; WA%: 0.775) and cluster 2 (T/D: 0.255; WA%: 0.75) (yellow arrows). PaO_2_: partial pressure of oxygen; PaCO_2_: partial pressure of carbon dioxide. FEV1: Forced expiratory volume in 1 second; FVC: Forced vital capacity; T/D ratio: ratio of airway wall thickness to total diameter; WA%, percentage of airway wall area to total area.

**A**
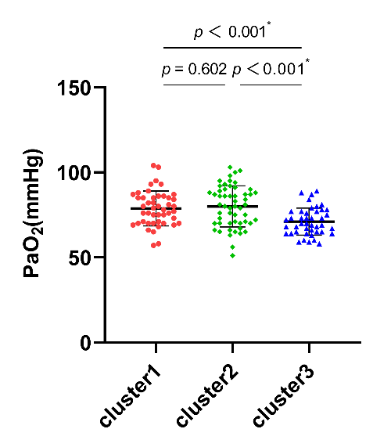
 **B**
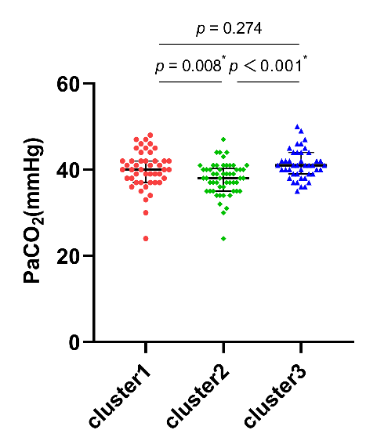


**C
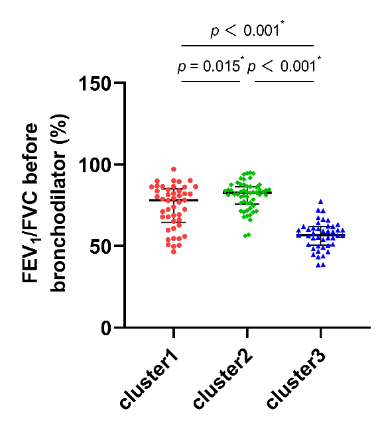
 D
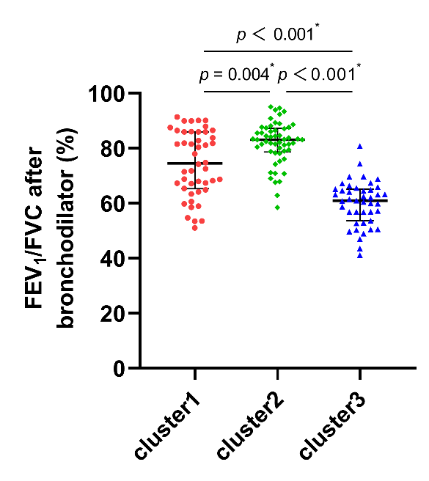
**

**E
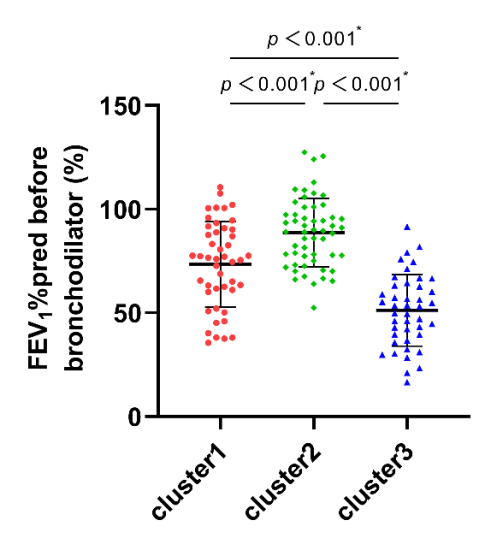
 F
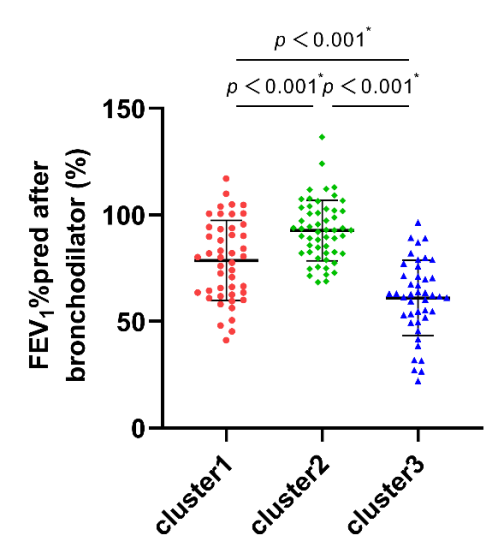
**

**G**
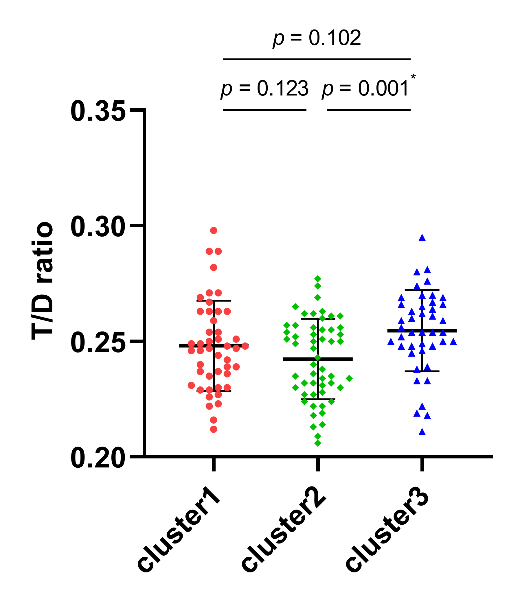
 **H**
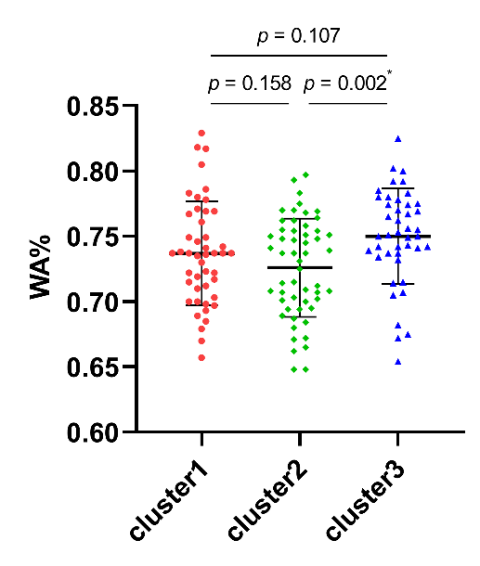


**
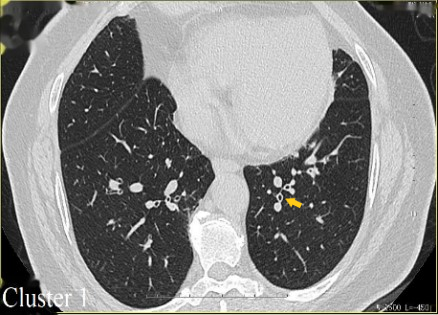

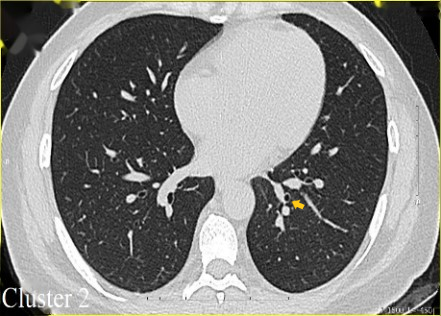

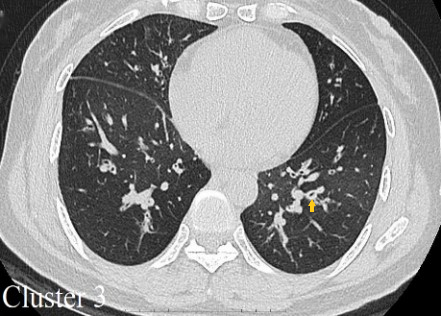
**

**I**

**Figure S3:** (A-E) Pairwise comparison of characteristics associated with inflammation type within the three clusters. T-IgE: total IgE; FeNO: Fractional exhaled nitric oxide; ppb: parts per billion. *statistical significance

**A**
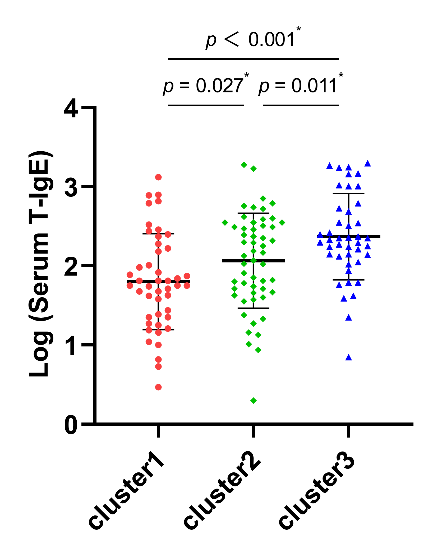
 **B**
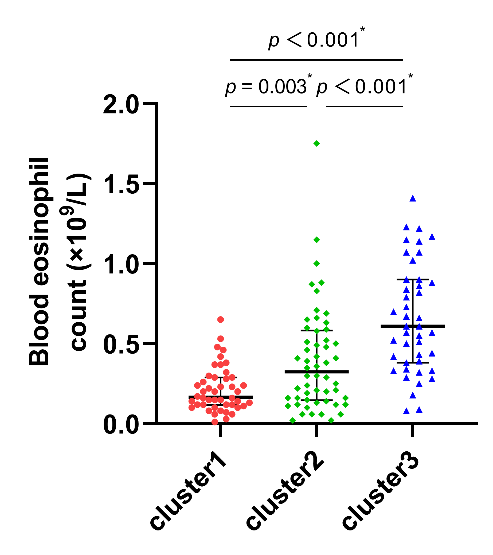


**C**
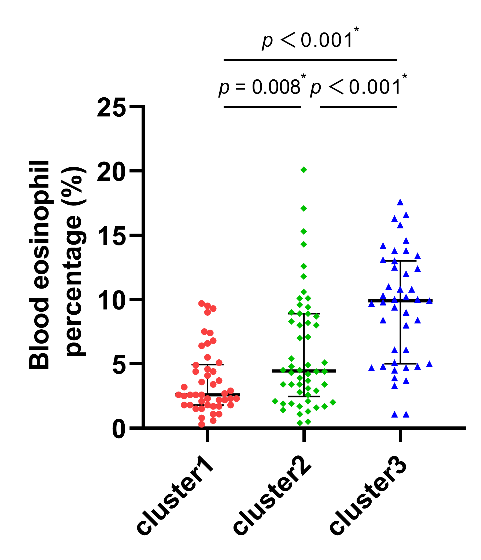
 **D**
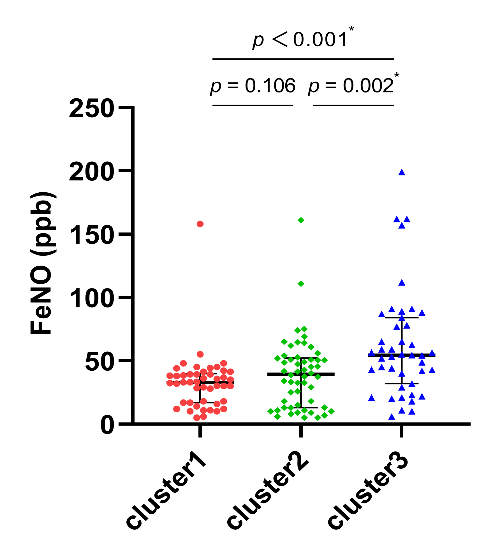


**E**
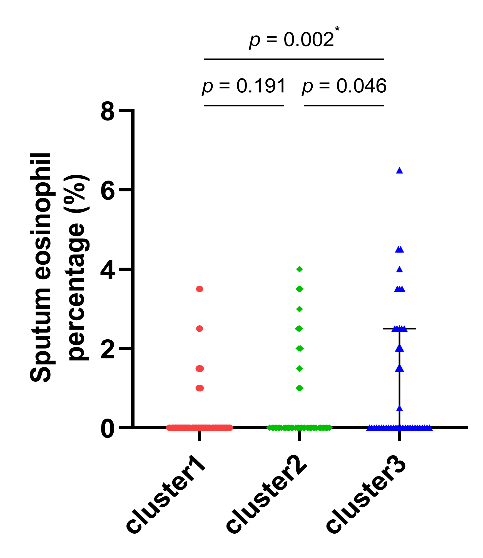

Supplement: Supplementary file 1 — Additional file 1. Figure S1: Comparison of characteristics associated with comorbid rhinosinusitis within the 3 clusters. [file 12931_2022_2028_MOESM1_ESM.docx]
